# Comparisons of oncological outcomes and perioperative complications between laparoscopic and open radical nephrectomies in patients with clinical T2 renal cell carcinoma (≥7cm)

**DOI:** 10.1371/journal.pone.0191786

**Published:** 2018-01-24

**Authors:** Hakmin Lee, Chung Un Lee, Jae Ho Yoo, Hyun Hwan Sung, Byong Chang Jeong, Seong Soo Jeon, Hyun Moo Lee, Han-Yong Choi, Chang Wook Jeong, Cheol Kwak, Seong Il Seo

**Affiliations:** 1 Department of Urology, Seoul National University Bundang Hospital, Seongnam, Republic of Korea; 2 Department of Urology, Samsung Medical Center, Sungkyunkwan University School of Medicine, Seoul, Republic of Korea; 3 Department of Urology, Seoul National University Hospital, Seoul, Republic of Korea; Cook County Hospital, UNITED STATES

## Abstract

**Purpose:**

Although minimal invasive techniques have been widely accepted in contemporary urology, the perioperative outcomes of laparoscopy in patients with clinical T2 renal cell carcinoma (RCC) have not been clearly evaluated. We aimed to compare the outcomes of laparoscopic radical nephrectomy (LRN) with those of open radical nephrectomy (ORN) in patients with clinical T2 RCC.

**Methods:**

We retrospectively analyzed the data of 835 patients who underwent radical nephrectomy for localized clinical T2 RCC (≥7 cm). The survival rates and postoperative complications were compared between the LRN and ORN groups. Multivariate Cox regression tests were performed to identify the independent predictors of each survival outcome.

**Results:**

There were 578 (69.2%) subjects in ORN group and 257 (30.8%) in LRN group, respectively. The LRN group showed a significant male predominance (p = 0.013), higher pathological stage (p = 0.02), and higher cellular grade (p = 0.010) compared with the ORN group. No significant differences in progression-free (p = 0.070), cancer-specific (p = 0.472), or overall survival (p = 0.249) were found between the two groups. In the multivariate analysis, the type of surgery did not show any significant associations with all three survival outcomes (all p > 0.2). Furthermore, there was no significant difference in postoperative complication rate between the two groups (p = 0.595). In the subgroup analysis according to tumor histology, no significant relationships were observed between survival outcome and surgery type.

**Conclusion:**

The LRN and ORN groups showed similar oncological outcomes in patients with clinical T2 RCC. Early postoperative complications were also comparable between LRN and ORN.

## Introduction

With the advent of modern imaging technology, more than 300,000 patients were diagnosed as having renal cell carcinoma (RCC) worldwide in 2012 [1]. The incidence of RCC has been constantly increasing and almost doubled in the past 2 decades in North America (7.6 per 100,000 person-years in 1988 to 11.7 per 100,000 person-years in 2006) [2]. Currently, the first choice of treatment for localized RCC is surgical removal by partial or radical nephrectomy [3]. Because of modern, advanced imaging modalities, an increased rate of incidental detection of small renal tumors has resulted in an overall downward stage migration [4–5]. Kane et al. showed that the increase in the incidence of RCC was mostly accounted for by an increase in organ-confined clinical-stage RCCs [4]. Mathew et al. also demonstrated global trends of an increasing incidence of RCCs after analyzing data from several worldwide databases [5]. However, about 25–30% of patients are found to have huge or metastatic disease at the time of diagnosis even with advances in imaging technology [6].

Laparoscopic radical nephrectomy (LRN) has been widely accepted by contemporary clinicians since the first laparoscopic total nephrectomy was performed at Washington University in 1990 [7]. Compared with traditional open radical nephrectomy (ORN), the laparoscopic approach has several advantages such as decreased blood loss, less postoperative narcotic requirements, and shorter hospital stay and duration of convalescence [8–10]. The guidelines from the European Association of Urology recommend LRN as the standard of care for patients with clinical T1 or T2 RCC not treatable with partial nephrectomy [11]. However, population-based studies have demonstrated that most radical nephrectomies were still performed by using the traditional open approach and only about 20% of LRN cases were performed in patients with large tumors (≥7 cm) [12]. Moreover, data on long-term oncological outcomes and operative feasibility are scarce and are based on the early adoptor’s small series and not on large-scale studies [13]. Therefore, we compared the long-term oncological outcomes and postoperative complications of LRN with those of ORN in a relatively large cohort that consisted of patients with clinical T2 RCC.

## Materials and methods

After obtaining ethical review board approval, we retrospectively reviewed the data of 964 patients with large renal tumors (≥ 7 cm) surgically treated with radical nephrectomy between 1994 and 2015 in two tertiary centers. After exclusion of patients with other malignancies, caval thrombus, distant metastasis, and benign pathology, we included 835 subjects in the analysis. Information on clinico-pathological outcomes was acquired from the prospectively maintained databases of the two institutions.

Routine preoperative radiological evaluation included abdominal computed tomography (CT), bone scans, and chest radiography (or chest CT). Tumor size was measured as the longest diameter of each tumor in any single plane of the preoperative imaging study. The laparoscopic nephrectomy was performed only by pure laparoscopic approach, not by hand or robot assisted techniques. The adrenal glands were routinely resected but sometimes preserved according to the physician’s clinical decision. Lymph node dissection was performed when there were suspicious findings indicating lymph node invasion in the preoperative imaging and/or intraoperative findings. Histological subtyping and pathologic staging were performed according to the 7^th^ edition of American Joint Committee guidelines and cellular grading was performed by the Fuhrman’s grading system [14–15]. Disease progression was defined as radiological or pathological evidence of local recurrence, distant metastasis, or mortality from RCC. Postoperative evaluations were slightly different across institutions and surgeons but were performed at 3- to 6-month intervals for the first 2 years and yearly thereafter. Mortality outcomes were acquired from the data of the Korean National Statistical Office and by a review of medical records. The early postoperative complications (within 30 days after surgery) were evaluated by central review of medical records for every subject included and classified by using the Clavien-Dindo system [16].

Independent *t* tests and chi-square tests were performed to analyze differences in clinicopathological characteristics according to the surgical approach. A Kaplan-Meier analysis with log-rank tests was used to evaluate survival differences after surgical management. A multivariate Cox proportional hazard model was used to identify the independent predictors of survival outcomes. All statistical analyses were performed by using SPSS 19.0 software (SPSS Inc., Chicago, IL, USA). All p values were two-sided, and p values < 0.05 were considered to indicate statistical significance.

## Results

The clinical and pathological characteristics of all of the patients are summarized in Table 1. For all patients, the median age was 56 years (interquartile range [IQR], 48–64 years); median tumor diameter, 9.0 cm (IQR 7.5–11.0 cm); and median follow-up time, 46.0 months (IQR 34–73 months). Among the patients, 578 underwent ORN and 257 underwent LRN. LRN was converted to ORN in 13 cases (because of severe bleeding, 6; other organ damage, 3; and technical difficulty, 4). Cases with open conversions were excluded from the analyses for the oncological outcomes but were included in the analysis of perioperative complications, as a part of the LRN group with grade III complications.

**Table 1:** Comparison of the clinicopathological characteristics of the entire patients who underwent radical nephrectomy, according to surgery type.

Parameters	Median (interquartile range) or counts (%)			p value
	Entire patients	ORN	LRN	
Number of subjects	835	578	257	
Age (years)	56 (48–64)	56 (47–64)	55 (49–64)	0.621
BMI (kg/m^2^)	24.0 (22.0–26.2)	23.9 (22.0–26.4)	24.2 (22.1–25.8)	0.139
Sex (male)	537 (64.3%)	362 (62.6%)	175 (68.1%)	0.013
ASA score (≥ 3)	459 (55.0%)	318 (55.0%)	141 (54.9%)	0.489
Diabetes mellitus (yes)	112 (13.4%)	90 (15.6%)	22 (8.6%)	0.010
Hypertension (yes)	262 (31.4%)	187 (33.9%)	75 (29.2%)	0.987
Tumor size (cm)	9.0 (7.5–11.0)	9.0 (8.0–11.0)	8.0 (7.0–9.1)	0.828
Pathological stage				0.028
pT1	96 (11.5%)	60 (10.4%)	35 (13.6%)	
pT2	414 (49.6%)	299 (51.7%)	115 (44.7%)	
pT3	310 (37.1%)	204 (35.3%)	106 (43.4%)	
pT4	16 (1.9%)	15 (2.6%)	1 (0.4%)	
Fuhrman grade				0.011
1	11 (1.3%)	9 (1.6%)	2 (0.8%)	
2	216 (25.9%)	168 (29.1%)	48 (18.7%)	
3	478 (57.2%)	313 (54.2%)	165 (64.2%)	
4	130 (15.6%)	88 (15.2%)	42 (17.2%)	
Histological subtype				0.063
Clear cell	672 (80.5%)	466 (80.6%)	206 (80.2%)	
Papillary	57 (6.8%)	40 (6.9%)	17 (6.6%)	
Chromophobe	86 (10.3%)	59 (10.2%)	27 (10.5%)	
Collecting duct	4 (0.5%)	2 (0.3%)	2 (0.8%)	
Unclassified	16 (1.9%)	11 (1.9%)	5 (2.0%)	
Estimated blood loss	250 (100–500)	200 (100–500)	300 (125–400)	0.381
Lymph node invasion	234 (28.0%)	176 (30.4%)	58 (22.6%)	0.544

ORN, open radical nephrectomy; LRN, laparoscopic radical nephrectomy; DM, diabetes mellitus; BMI, body mass index; ASA, American Society of Anesthesiologists; SMD, standardized mean differences

When we compared clinical characteristics according to surgery type, the LRN group had a lower prevalence of diabetes mellitus (p = 0.014) and male predominance (p = 0.013) but no significant difference in age (p = 0.621), body mass index (BMI; p = 0.139), and tumor size (p = 0.828) compared with the ORN group. The LRN group had worse pathological profiles than the ORN group in terms of higher pathological stage (p = 0.027) and cellular grade (p = 0.010).

**Fig 1:**
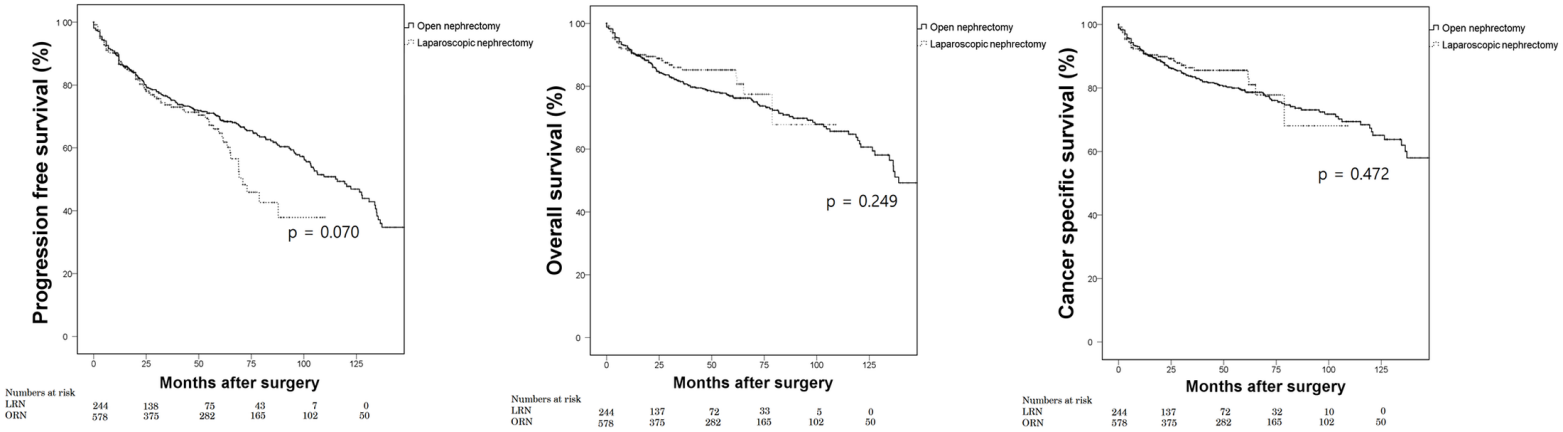
Kaplan-Meier analysis of progression-free, overall, and cancer-specific survival according to surgery type.

After a median of 22.5 months (IQR, 7.1–60.0 months) after surgery, 286 patients developed disease progression. A total of 159 cancer-specific mortalities after a median of 17.0 months (IQR, 6.0–42.5) and 179 overall mortalities after a median of 20 months (IQR 6–47.8 months) were observed. When we compared the survival outcomes between the ORN and LRN groups by using Kaplan-Meier analyses, we found no significant differences in progression-free survival (PFS; p = 0.070), overall survival (OS; p = 249), or cancer-specific survival (CSS; p = 0.472; Fig 1). Subsequently, multivariate Cox analyses were performed to identify significant prognostic predictors of each survival end point. Older age, male sex, low BMI, and large tumor size were identified as significant adverse prognostic factors of PFS, OS, and CSS (Table 2). In contrast, surgery type did not show any significant association with postoperative survival outcomes.

**Table 2:** Multivariate analysis using the Cox proportional hazard model of possible predictors of progression-free, cancer-specific, and overall survival after radical nephrectomy in patients with large (≥ 7 cm) non-metastatic renal cell carcinoma.

	Progression-free survival			Overall survival			Cancer-specific survival		
	HR	95% CI	p value	HR	95% CI	p value	HR	95% CI	p value
Age	1.015	1.004–1.028	0.011	1.032	1.018–1.047	< 0.001	1.025	1.010–1.041	0.001
BMI	0.897	0.862–0.934	< 0.001	0.868	0.826–0.913	< 0.001	0.857	0.812–0.904	< 0.001
Sex (female)	0.622	0.471–0.822	0.001	0.629	0.450–0.879	0.007	0.591	0.412–0.848	0.004
Diabetes mellitus (yes)	1.175	0.843–1.638	0.340	1.448	0.990–2.118	0.057	1.519	1.018–2.268	0.041
Hypertension (yes)	1.440	1.089–1.905	0.010	1.364	0.971–1.914	0.073	1.363	0.949–1.957	0.093
ASA score (≥ 3)	0.858	0.671–1.098	0.223	0.837	0.619–1.131	0.247	0.903	0.654–1.246	0.533
Tumor size	1.128	1.081–1.177	< 0.001	1.167	1.113–1.223	< 0.001	1.175	1.119–1.233	< 0.001
Fuhrman grade (≥ 3)	0.856	0.665–1.103	0.230	0.796	0.584–1.085	0.149	0.723	0.521–1.003	0.052
T stage (≥ 3)	1.167	0.864–1.577	0.314	1.404	0.990–1.990	0.057	1.396	0.962–2.026	0.079
Lymph node invasion (yes)	1.068	0.822–1.388	0.621	1.049	0.761–1.445	0.771	1.115	0.795–1.563	0.529
Surgery type (laparoscopy)	1.178	0.881–1.575	0.270	0.825	0.561–1.211	0.325	0.873	0.589–1.296	0.501
Cellular type (non-clear cell)	0.589	0.416–0.834	0.003	0.829	0.558–1.232	0.354	0.717	0.462–1.110	0.136

ASA, American Society of Anesthesiologists; BMI, body mass index; ECOG, Eastern Cooperative Oncology Group; HR, hazard ratio; CI, confidence interval

Survival outcomes between the LRN and ORN groups were compared separately in each subgroups according to the pathologic stage of RCC. When we performed Kaplan-Meier analysis of the 496 patients with pathologic stage of pT2, we found no significant difference between the LRN and ORN groups in terms of PFS (p = 0.984), OS (p = 0.186), or CSS (p = 0.243). Further analyses of the 326 patients with the higher pathologic stage (≥pT3) also did not show any significant difference in PFS (p = 0.111), OS (p = 0.427), or CSS (p = 0.690). Multivariate Cox proportional analyses also showed no significant relationships between surgical approach and survival outcomes in subgroups according to the pathologic stage (Table 3). Furthermore, we compared postoperative complication rates between the LRN and ORN groups and the complication rates were not significantly different between the two groups (p = 0.595; Table 4).

**Table 3:** Multivariate analyses using the Cox proportional hazard model of the association of surgical type (laparoscopic versus open nephrectomy) with survival outcomes.

		HR (95% CI)	p value
All RCCs	n = 822		
	Recurrence	1.195 (0.894–1.597)	0.230
	Overall mortality	0.756 (0.492–1.162)	0.203
	Cancer-specific mortality	0.794 (0.510–1.234)	0.305
pT2	n = 496		
	Recurrence	1.001 (0.612–1.639)	0.996
	Overall mortality	0.678 (0.342–1.347)	0.268
	Cancer-specific mortality	0.712 (0.328–1.412)	0.292
≥ pT3	n = 326		
	Recurrence	1.161 (0.831–1.622)	0.381
	Overall mortality	0.908 (0.607–1.356)	0.169
	Cancer-specific mortality	0.802 (0.485–1.260)	0.312

RCC, renal cell carcinoma; HR, hazard ratio; CI, confidence interval

All multivariate analyses were adjusted for the following variables: age, sex, body mass index, American Society of Anesthesiologists score, T stage (not included in the subgroup analyses), tumor size, Fuhrman grade, and cellular type.

**Table 4:** Differences between postoperative complications according to surgical approach in the patients with large renal cell carcinoma (≥ 7 cm) treated with radical nephrectomy.

Clavien-Dindo classification	ORN group	LRN group	p value
	Counts (%)		
No complication	512 (88.6)	226 (87.9)	0.595*
Grade I	38 (6.6)	16 (6.2)	
Grade II	11 (1.9)	0 (0.0)	
Grade III	12 (2.1)	15 (5.8)	
Grade IV or higher	5 (0.9)	0 (0.0)	

ORN, open radical nephrectomy; LRN, laparoscopic radical nephrectomy

* p values were calculated by chi-square test comparing the grade ≤ 1 versus ≥ 2.

## Discussion

Even though several previous study groups showed the feasibility of laparoscopic surgery in patients with large renal tumors [17–19], ORN remains the first choice of treatment for patients with large renal cell carcinomas [12]. The preference for ORN might be because of several reasons. The technical difficulties in handling a huge kidney mass inside the peritoneal space are probably the primary reason. These difficulties include a cramped working space, poor visualization due to a large specimen, increased vascularity around the kidney, and difficult specimen control, which may be connected to several catastrophic accidents [13]. These difficulties might discourage surgeons, even experienced endourologists, from performing LRN.

So far, most of the studies conducted on LRN were confined to clinical T1 disease and only a few studies addressed the clinical role of LRN in patients with clinical T2 RCCs [13, 17–19]. However, these studies were also limited by the small numbers of subjects and relatively short-term follow-up periods. To our best knowledge, the present study is the largest among the studies that compared oncological outcomes between ORN and LRN that had a relatively long follow-up period.

Steinberg et al. previously compared the postoperative outcomes of LRN and those of ORN and found that clinical T2 renal masses can be efficiently managed by using the laparoscopic approach, with additional advantages of a shorter hospital stay, decreased blood loss, and more rapid recovery than ORN [18]. When they compared the outcomes of LRN according to tumor size (≥7 vs. <7 cm), they found no significant difference in complication rates between the two groups. In another study, Hemal et al. also compared postoperative outcomes between LRN and ORN in patients with clinical T2 renal tumors but with a longer follow-up period [19]. They found that the LRN group had superior postoperative outcomes in terms of less blood loss, shorter hospital stay, decreased analgesic requirement, and more rapid convalescence even though the LRN group required a longer operation time than the ORN group (180.8 vs. 165.3 minutes, p = 0.029). More recently, Pierorazio et al. retrospectively analyzed the perioperative and oncological outcomes of 200 patients with clinical T2 renal tumors [20]. They observed higher open conversion rates and blood loss in patients with larger tumors and reported an overall open conversion rate of 5% in their cohort.

Previous studies demonstrated comparable oncological outcomes with LRN, but performing LRN for large renal tumors is still difficult even under experienced hands. Pierorazio et al. showed evidence that laparoscopic surgery is more dangerous to perform for larger tumors although they concluded that LRN for clinical T2 tumors is feasible [20]. When they compared the perioperative outcomes according to tumor size, the patients with large tumors had longer operating times, greater amounts of blood loss, and a significantly higher open conversion rate (14%) than patients with small tumors. We experienced a similar open conversion rate of 5.1% when performing LRN compared with that reported for previous studies [19–20]. In the present study, we did not observe any significant differences in progression-free survival, cancer-specific survival, or OS between the LRN and ORN groups, reinforcing the reports of previous studies that oncological outcomes are similar after LRN and ORN in patients with clinical T2 RCC.

In the present study, all of the LRNs were performed by the transperitoneal approach even if most of the participating surgeons had sufficient experience in performing the retroperitoneal approach. Compared with the retroperitoneal approach, the transperitoneal approach has more advantages in dealing with large specimens because it enables securing a more sufficient working space. In our study, some surgeons performed preoperative selective embolization of the renal artery in some highly challenging cases to reduce the incidence of intraoperative morbidities.

In our study, the complication free rates were 88.6% and 87.6% for ORN and LRN, respectively, when we consider the cases with open conversions to be grade III complications of the LRN group. Some may argue against open conversion being classified as an intraoperative complication. However, when we compared the complications of the ORN and LRN groups after exclusion of the open conversion cases, there were no significant differences in the overall complication rates between the two groups (p = 0.163).

Previous studies reported relatively higher overall complication rates, ranging from 12% to 22.3%, than in the present study after LRN in patients with clinical T2 renal tumors [20–21]. The relative low complication rates of the present study may be due to the fact that a high percentage of the patients in our cohort routinely used a patient-controlled self-analgesic and anti-emetic administrating system, which may lower the rate of mild complications such as wound pain and nausea compared with the previous studies of other groups.

Our study has some limitations. Due to its retrospective cross-sectional design, an inherent structural bias may exist. Moreover, the patients in this study received different salvage therapies according to the preference of clinicians. This heterogeneity might have affected the survival outcomes in our study. A more important limitation is that we could not analyze the impact of anatomical tumor location, which is known to be an independent prognostic factor. Because the surgical approach was decided by each surgeon according to their individual criteria, the challenging cases with tough anatomy, including centrally located tumors and huge hilar tumors, may have been underrepresented in the LRN group which causing possible selection bias in the present study. Furthermore, present study could not analyze several variables which is traditionally used to highlight the minimal invasive surgery upon conventional open approach such as recovery time, convalescence periods and et cetera, which is also one of our main limitations. Therefore, a large prospective randomized study is needed to determine the oncological and perioperative outcomes of LRN for large clinical T2 renal tumors.

## Conclusion

LRN and ORN were equally effective in terms of oncological outcomes. The overall complication rates were comparable between the two surgical techniques. LRN should be considered as a valuable treatment option for patients with clinical T2 RCCs.

## Clinical practice points

The laparoscopic radical nephrectomy for large renal cell carcinoma (≥7cm) has not been clearly evaluated. We compared the open and laparoscopic radical nephrectomy in the largest cohorts ever evaluated. Laparoscopic radical nephrectomy showed equivalent oncological outcomes and postoperative complications compared with open radical nephrectomy. The indication of laparoscopic nephrectomy can be carefully expanded to large RCC over diameter of 7 centimeters.

## Abbreviations

BMI: Body mass index
CSS: Cancer specific survival
CT: Computed tomography
HR: Hazard ratio
IQR: Interquartile range
LRN: Laparoscopic radical nephrectomy
ORN: Open radical nephrectomy
OS: Overall survival
PFS: Progression-free survival
RCC: Renal cell carcinoma
